# *De-novo* genome assembly and annotation of sobaity seabream *Sparidentex hasta*

**DOI:** 10.3389/fgene.2022.988488

**Published:** 2022-10-31

**Authors:** Qusaie Karam, Vinod Kumar, Anisha B. Shajan, Sabeeka Al-Nuaimi, Zainab Sattari, Saleem El-Dakour

**Affiliations:** ^1^ Crises Management and Decision Support Program, Environment and Life Sciences Research Center, Kuwait Institute for Scientific Research, Kuwait City, Kuwait; ^2^ Biotechnology Program, Environment and Life Sciences Research Center, Kuwait Institute ForScientific Research, Kuwait City, Kuwait; ^3^ Aquaculture Program, Environment and Life Sciences Research Center, Kuwait Institute ForScientific Research, Kuwait City, Kuwait

**Keywords:** draft genome, fisheries and aquaculture, food security, Kuwait, assembly and annotation

## Abstract

*Sparidentex* *hasta* (Valenciennes, 1830) of the Sparidae family, is an economically important fish species. However, the genomic studies on *S. hasta* are limited due to the absence of its complete genome. The goal of the current study was to sequence, assemble, and annotate the genome of *S. hasta* that will fuel further research related to this seabream. The assembled draft genome of *S. hasta* was 686 Mb with an N50 of 80 Kb. The draft genome contained approximately 22% repeats, and 41,201 genes coding for 44,555 transcripts. Furthermore, the assessment of the assembly completeness was estimated based on the detection of ∼93% BUSCOs at the protein level and alignment of >99% of the filtered reads to the assembled genome. Around 68% of the predicted proteins (*n* = 30,545) had significant BLAST matches, and 30,473 and 13,244 sequences were mapped to Gene Ontology annotations and different enzyme classes, respectively. The comparative genomics analysis indicated *S. hasta* to be closely related to *Acanthopagrus latus*. The current assembly provides a solid foundation for future population and conservation studies of *S. hasta* as well as for investigations of environmental adaptation in Sparidae family of fishes. Value of the Data: This draft genome of *S. hasta* would be very applicable for molecular characterization, gene expression studies, and to address various problems associated with pathogen-associated immune response, climate adaptability, and comparative genomics. The accessibility of the draft genome sequence would be useful in understanding the pathways and functions at the molecular level, which may further help in improving the economic value and their conservation.

## Introduction

Sobaity seabream, *Sparidentex hasta* (Valenciennes, 1830) belongs to the Sparidae family, which comprises 35 genera, 132 species, and 10 subspecies (de la Herran et al., 2001). The species has a wide geographic distribution extending from the Arabian Gulf to the sea of Oman and the western Indian Ocean, and the Indian coasts (Carpenter et al., 1997). *S. hasta* is recognized as one of the most promising species for aquaculture, because of its good adaptation to captivity, rapid growth, and high market price. Further, it is of high economic significance in Kuwait and the Arabian Gulf regions.

The anthropogenic and fishing activities around the coastal regions are affecting marine fauna including the population of many commercially important fish species (Bukola et al., 2015). However, genomics and molecular biology research on Sobaity seabream is limited due to the absence of its complete genome sequence. The DNA barcoding of several commercial seabreams including *S. hasta* was reported (Al-Zaidan et al., 2021). Most of the other studies on *S. hasta* are focused on the dietary effects of different feed combinations on *S. hasta* (Hossain et al., 2017; Yaghoubi et al., 2018; Hekmatpour et al., 2019). Furthermore, a study on the response of *S. hasta* larvae to the toxicity of dispersed and undispersed crude oil was reported (Karam et al., 2021).

There is an increasing demand for fish in Kuwait as fisheries only fulfill about 30% of local fish demand, as the other 70% is met through imports. However, there is a global decline in fisheries (Hossain et al., 2017) and to compensate for this decline and to assure future food security in Kuwait, aquaculture technologies were developed for *S. hasta* to fulfill the demands of the local market (Teng et al., 1987; Abdullah et al., 1989). Sobaity was chosen to be the first candidate species for commercial production in Kuwait because of its survival capability and tolerance in captivity (Al-Abdul-Elah et al., 2010; Torfi Mozanzadeh et al., 2017) and it is the second most favorable commercial seabream species in Kuwait after the yellowfin seabream (*Acanthopagrus latus*) (Al-Zaidan et al., 2021). The selection of Sobaity for aquaculture in the early 80’s was primarily attributed to its ability to spawn in captivity, its tolerance to different culture conditions, and its fast growth rate (Yousif et al., 2003). *S. hasta* can exercise a wide range of tolerance to changes in water quality parameters such as dissolved oxygen, temperature, pH, and salinity which are reflective of natural ambient conditions. However, extreme and abrupt changes in those environmental parameters can result in the deteriorating health of juvenile Sobaity in culture tanks (European Food Safety Authority, 2008; Zainal and Altuama, 2020).

Also, Sobaity is sought for its nutritional value as a healthy seafood commodity. Fishes containing a certain type of fatty acids are known to reduce the risk of coronary heart disease (Kris-Etherton et al., 2002). In particular, Sobaity is rich in n-3 polyunsaturated fatty acids (PUFA), docosahexaenoic acid (DHA), and eicosapentaenoic acid (EPA). Interestingly, the wild-caught Sobaity contains a higher n-3 PUFA than their cultured counterparts (Hossain et al., 2017; Hossain et al., 2019). Moreover, the highest muscle lipid content recorded for Sobaity was during the pre-spawning and spawning seasons.

An extinction risk assessment of marine fishes, mainly for seabreams, conducted recently based on the dataset of the International Union for Conservation of Nature’s Red List indicated that around 25 species are in threatened/near-threatened condition as shown by their body weight (Comeros-Raynal et al., 2016). In this context, the availability of the complete genome sequence may help in understanding the detailed pathways and functions at the molecular level, which may further help in improving the economic value of the fish as well as pave better ways for their conservation.

Next-generation sequencing has propelled the construction of draft genome sequences of various important organisms (Goodwin et al., 2016) including many fish species from the Sparidae family (Shin et al., 2018; Zhu et al., 2021). The complete genome sequence is available for very few species of Sparidae family that include *Sparus aurata* (Pauletto et al., 2018), *Spondyliosoma cantharus* (GCA_900302685), *Pagrus major* (Shin et al., 2018), and the most recent one *Acanthopagrus latus* (Zhu et al., 2021). The genome of *S. aurata* is approximately 830 Mb and had a GC content of 42% (GCA_900880675.2). The genome of *P. major* is ∼875 Mb with a GC content of 38%. The draft genome contained a total of 886,260 scaffolds with an N50 of 4.6 Mb (GCA_002897255.1). *S. cantharus* genome is approximately 680 Mb in length containing 47,064 scaffolds (GCA_900302685.1), whereas the size of *A. latus* genome (GCF_904848185.1) is ∼685 Mb contained within 66 scaffolds. The study on *A. latus* presented a chromosome-level genome assembly and explored the molecular basis of sex reversal and the characteristics of the osmoregulation in this species (Zhu et al., 2021).

In the current study, our goal was to sequence, assemble, and annotate the draft genome of *S. hasta*. The draft genome assembly will facilitate future investigations of the biology of this species and provide a valuable resource for the conservation and breeding management of *S. hasta*.

## Materials and methods

### DNA isolation from fin tissues of sobaity fish

The genomic DNA was isolated from 4 months old female Sobaity fish collected from the Mariculture and Fisheries Department, Kuwait Institute for Scientific Research, Salmiya, Kuwait. DNA isolation was performed from the fin tissues (80 mg) using GenElute Plant Genomic DNA Miniprep Kit. The quantity of the genomic DNA was estimated using a Nanodrop spectrophotometer and Qubit fluorometer 3.0, and quality was checked by the A260/280 value and 0.8% agarose gel electrophoresis.

### RNA isolation

The sobaity seabream larvae were reared in aerated tanks with six air stones, illuminated by natural sunlight and fluorescent light (40 W) with 1,500 lux light intensity in the day and 1,000 lux at night time (Al-Abdul-Elah, 1984; Teng et al., 1999). The stock density in *S. hasta* rearing tanks was 40 larvae/L seawater. The 24 h post-hatch larvae were transferred from Mariculture and Fisheries Department and acclimated to laboratory conditions at the Ecotoxicology Laboratory, Kuwait Institute for Scientific Research, Kuwait. Total RNA from 100 mg of the larvae was extracted using the Invitrogen TRIzol reagent (Life Technologies Corporation, United States) following the instructions provided by the manufacturer. Genomic DNA contamination in the extracted RNA samples was removed using the On-Column DNase 1 Digestion Set (DNASE70, Sigma-Aldrich, United States).

### Library preparation and sequencing

One microgram of genomic DNA was randomly fragmented by Covaris. The fragmented genomic DNA was selected by Agencourt AMPure XP-Medium kit to an average size of 200–400 bp. Fragments were end-repaired and then 3’ adenylated. Adaptors were ligated to the ends of these 3’ adenylated fragments and the fragments were amplified using PCR. The PCR products were purified by the Agencourt AMPure XP-Medium kit. The double-stranded PCR products were heat denatured and circularized by the splint oligo sequence. The single-strand circle DNA (ssCir DNA) were considered as the final library. The library was validated on the Agilent Technologies 2100 bioanalyzer. The qualified libraries were sequenced by BGISEQ-500: ssCir DNA molecule formed a DNA nanoball (DNB) containing more than 300 copies through rolling-cycle replication. The DNBs were loaded into the patterned nanoarray by using a high-density DNA nanochip technology. Finally, 150 bp pair-end reads were obtained by combinatorial Probe-Anchor Synthesis (cPAS). The next-generation sequencing was performed at BGI, Hongkong.

### *De-novo* genome assembly

The high-quality paired-end DNA sequencing data was used for *de novo* assembly of the *S. hasta* genome using MaSuRCA-4.0.3 (Zimin et al., 2013). The MaSuRCA assembler combines the benefits of *deBruijn* graph and Overlap-Layout-Consensus assembly approaches. It aids in different types of analysis by integrating various tools for genome size estimation, error correction, assembly scaffolding, polishing, and has been widely used by the scientific community. In addition, MaSuRCA has been suggested to be at least equal to or better than most of the genome assemblers in terms of assembly quality and completeness by the comparative studies performed on eukaryotic genomes (Mikheenko et al., 2018; Sohn and Nam, 2018). The paired-end reads were error corrected using QuorUM (Marçais et al., 2015), and then used for the construction of k-unitigs. Further, the paired-end reads were extended to form super reads with the help of unitigs. After creating the super-reads, contiging and scaffolding was performed using a modified version of CABOG assembler. Finally, gaps in the scaffold assembly were filled. All the steps were performed using the MaSuRCA assembler. The genome size was estimated using the jellyfish mer-counter, integrated within MaSuRCA. Additionally, we have used backmap tool (Schell et al., 2017; Pfenninger et al., 2022) which estimates the genome size based on the reads mapped to the assembly. The primary assembly was filtered to remove scaffolds shorter than 500 bp.

### Repeat annotation and masking

A *de novo* repeat library for *S. hasta* filtered assembly was constructed using RepeatModeler (Flynn et al., 2020), which employs three repeat-finding methods; RECON (Bao and Eddy, 2002), RepeatScout (Price et al., 2005), and TRF (Benson, 1999). The repeat library was then subjected to RepeatMasker to find and mask the repeats in the assembled genome using RMBlast as the default search engine.

### Gene prediction, annotation, and assembly completeness

The BRAKER2 pipeline (Brůna et al., 2021) was used to perform gene prediction by integrating *ab initio* gene prediction, RNA-seq based prediction, and predictions based on vertebral protein sequences, which combined the advantages of both GeneMark-ET and AUGUSTUS. The RNA-seq data was generated from the post-hatch fish larvae of Sobaity-seabream (BioProject Accession: PRJNA748027). The filtered RNA-seq reads were aligned to the repeat masked assembly using TopHat2 (Kim et al., 2013) with default parameters. The vertebral protein sequences from various species (*n* = 4,937,339) used for gene prediction were downloaded from the OrthoDB database (Kriventseva et al., 2019). The ProtHint (Brůna et al., 2020) protein mapping pipeline was used for generating required hints from the vertebral protein sequences for BRAKER. The assembled scaffolds along with the aligned reads (BAM files) and generated hints from the protein sequences were used for generating initial gene structures using the GeneMark-ET tool (Lomsadze et al., 2014). The initial gene structures were then used for training by AUGUSTUS to produce the final gene predictions (Stanke and Waack, 2003). The predicted genes were submitted to Blast2GO tool (Conesa et al., 2005) for annotation.

The raw reads were aligned back to the filtered scaffolds to assess the quality of the genome assembly using Bowtie 2 (Langmead and Salzberg, 2012). Furthermore, the predicted genes from the BRAKER2 pipeline were subjected to BUSCO version 5.2.2 (Manni et al., 2021) to evaluate the completeness of the assembled genome, based on the vertebrata_odb10 database.

### Comparative genomics and phylogenetic analysis

We used OrthoMCL v2.0.9 (Li et al., 2003) for ortholog analysis based on protein datasets from the BRAKER2 pipeline and four other fish species: *Diplodus sargus* (txid: 38,941), *Spondyliosoma cantharus* (taxid: 50,595), *Sparus aurata* (txid: 8175), *Acanthopagrus latus* (txid: 8177). For *S. aurata* (GCA_900880675.1) and *A. latus* (GCA_904848185.1), the protein sequences were downloaded from the RefSeq database and used for the phylogenetic analysis. However, for *D. sargus* (GCA_903131615.1) and *S. cantharus* (GCA_900302685.1), the genome sequences were downloaded from the NCBI and proteins were predicted using BRAKER2 pipeline. These protein sequences were then used for the phylogenetic analysis. CD-HIT (Li and GodzikCd-hit, 2006) was used to remove redundant sequences (≥90% identity) in each organism. The protein sequences were further filtered to remove poor quality sequences using ‘orthomclFilterFasta’ command using default parameters. Then, the non-redundant filtered protein sequences were subjected to all-against-all BLASTp (Altschul et al., 1990) with an E-value of 1e-5. The blast results were used to identify single-copy orthologs using OrthoMCL across the species. The single copy ortholog sequences were then used for multiple sequence alignment by MAFFT, the result of which was used for the construction of phylogenetic tree using FastTree (Price et al., 2009).

## Results

### Draft genome assembly of *S. hasta*

**FIGURE 1:**
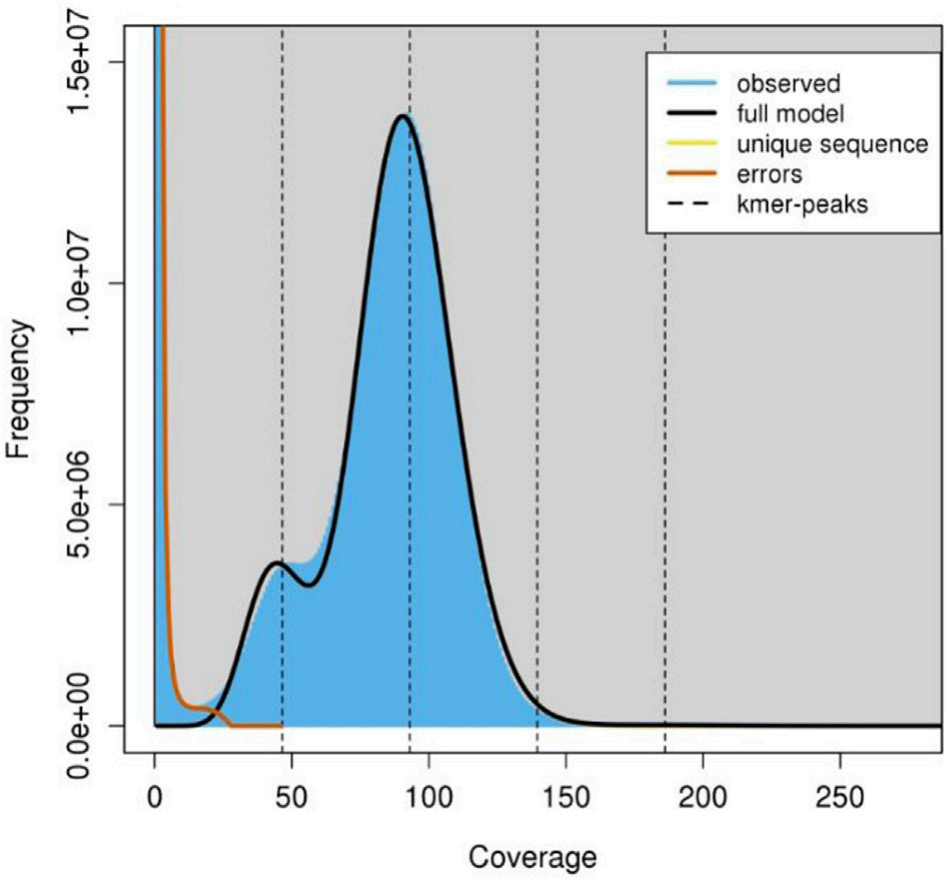
K-mer frequency coverage. The graph indicates the frequency of observed kmers using which the genome size is estimated. The jellyfish histo file was used as the input and the graph was constructed using the GenomeScope tool (http://qb.cshl.edu/genomescope/).

A total of approximately 550 million paired-end reads were used for constructing the genome assembly of *S. hasta*. The genome size of *S. hasta* was estimated to be around 703 Mb based on *k*-mer statistics using jellyfish *k*-mer counter (Figure 1) integrated within MaSuRCA and 688.8 Mb based on backmap tool. The slight difference in the estimated genome size by both the tools could be attributed to the different approaches used by these tools. The size of the assembled genome was ∼687 Mb contained within 22,741 scaffolds. The assembly was filtered to remove the scaffolds shorter than 500 Mb, and the final filtered assembly contained 20,442 scaffolds. The size of the filtered assembly was ∼686 Mb. The final assembly contained very low N content (∼0.04%). Furthermore, the alignment of the cleaned reads indicated successful matching of 99% of the raw reads back to the filtered assembly, suggesting the completeness of the assembly. The filtered assembly was used for further analysis. The complete statistics of the filtered assembly is provided in Table 1.

**TABLE 1:** Statistics of the final filtered assembly.

No. of scaffolds	20,442
GC-content	42.1%
L50	2,427
L90	9,117
N50 (bp)	80,670
N90 (bp)	18,310
Min. length	500
Max. length	770,404
Mean length	33,588
Median length	14,545
No. of bases	686609404
No. of ‘As’	198736919
No. of ‘Cs’	144604718
No. of ‘Gs’	144492944
No. of ‘Ts’	198522703
No. of ‘Ns’	252,120

The L50, L90, N50 and N90 statistics indicate the assembly quality. L50 and L90: Count of smallest number of contigs whose length sum makes up 50% and 90% of the genome size, respectively. N50 and N90: 50% and 90% of the entire assembly is contained in scaffolds that are equal to or larger than these values.

### Repeat identification, annotation, and masking

The repeat sequences in the filtered assembly were predicted by RepeatModeller and masked using RepeatMasker. The total length of repetitive sequences was ∼153.4 Mb, accounting for ∼22.35% of the draft genome size. Among these, ∼12% of the repeats were unclassified. DNA transposons corresponded to ∼5%, whereas retroelements corresponded to ∼2% of the genome. A complete list of different repeats along with their content in the draft genome has been shown in Table 2.

**TABLE 2:** Repeat annotation of the assembly.

Type of repeats		Number of elements	Length (bp)	% of sequence
Retroelements		59,487	13902715	2.02
	SINEs	6,590	859,207	0.13
	Penelope	3,800	518,117	0.08
	LINEs	42,621	10345032	1.51
	L2/CR1/Rex	29,189	6459372	0.94
	R1/LOA/Jockey	2,317	327,028	0.05
	R2/R4/NeSL	152	87,166	0.01
	RTE/Bov-B	5,405	1978982	0.29
	L1/CIN4	1,310	681,130	0.1
	LTR elements	10,276	2698476	0.39
	BEL/Pao	612	282,964	0.04
	Gypsy/DIRS1	2,343	1199000	0.17
	Retroviral	3,156	479,547	0.07
DNA transposons		213,033	34752653	5.06
	hobo-Activator	97,375	15466145	2.25
	Tc1-IS630-Pogo	29,790	5793811	0.84
	PiggyBac	3,453	383,971	0.06
	Tourist/Harbinger	9,907	2052324	0.3
	Other (Mirage, P-element, Transib)	2,563	498,544	0.07
Unclassified		547,666	82948695	12.08
Total interspersed repeats			131604063	19.17
Rolling-circles		12,167	2543620	0.37
Small RNA		4,738	772,793	0.11
Satellites		3,281	493,762	0.07
Simple repeats		383,453	16415048	2.39
Low complexity		41,575	2219709	0.32

SINE: Short interspersed nuclear element; LINE: Long interspersed nuclear element; LTR: Long terminal repeat.

### Gene prediction and annotation of the draft assembly

**FIGURE 2:**
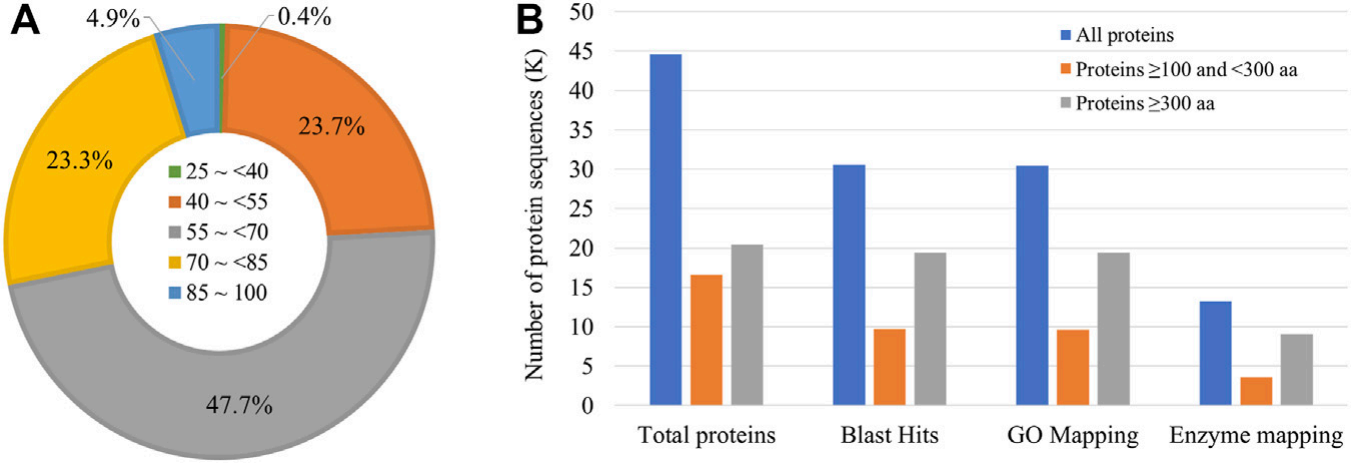
Annotation of *Sparidentex* hasta protein sequences. **(A)** Doughnut chart indicating the percentage of sequences with different blast similarity means. **(B)** Annotation of the protein sequences. Different coloured bars indicate the annotation of proteins of varying lengths.

Gene prediction using BRAKER2 pipeline based on *ab-initio* method, and RNA-seq and ortholog protein sequence-based prediction resulted in a total of 41,201 genes coding for 44,555 transcripts. The mean length of the coding sequence was 1,249 bp, whereas that of protein sequence was 416 aa. Among the protein sequences, a total of 30,545 sequences (68.5% of all the protein sequences) had significant BLAST matches. Many sequences had a mean similarity score of more than 50 (Figure 2A).

Furthermore, 30,473 and 13,244 sequences were mapped to Gene Ontology annotations and different enzyme classes, respectively (Figure 2B). The assessment of the assembly completeness indicated the detection of 93.4% complete BUSCOs (Benchmarking Universal Single-Copy Orthologs) at the protein level, with the single-copy, duplicated, fragmented, and missing accounting for 82.8, 10.6, 5.1, and 1.5%, respectively.

### Comparative genomics

**FIGURE 3:**
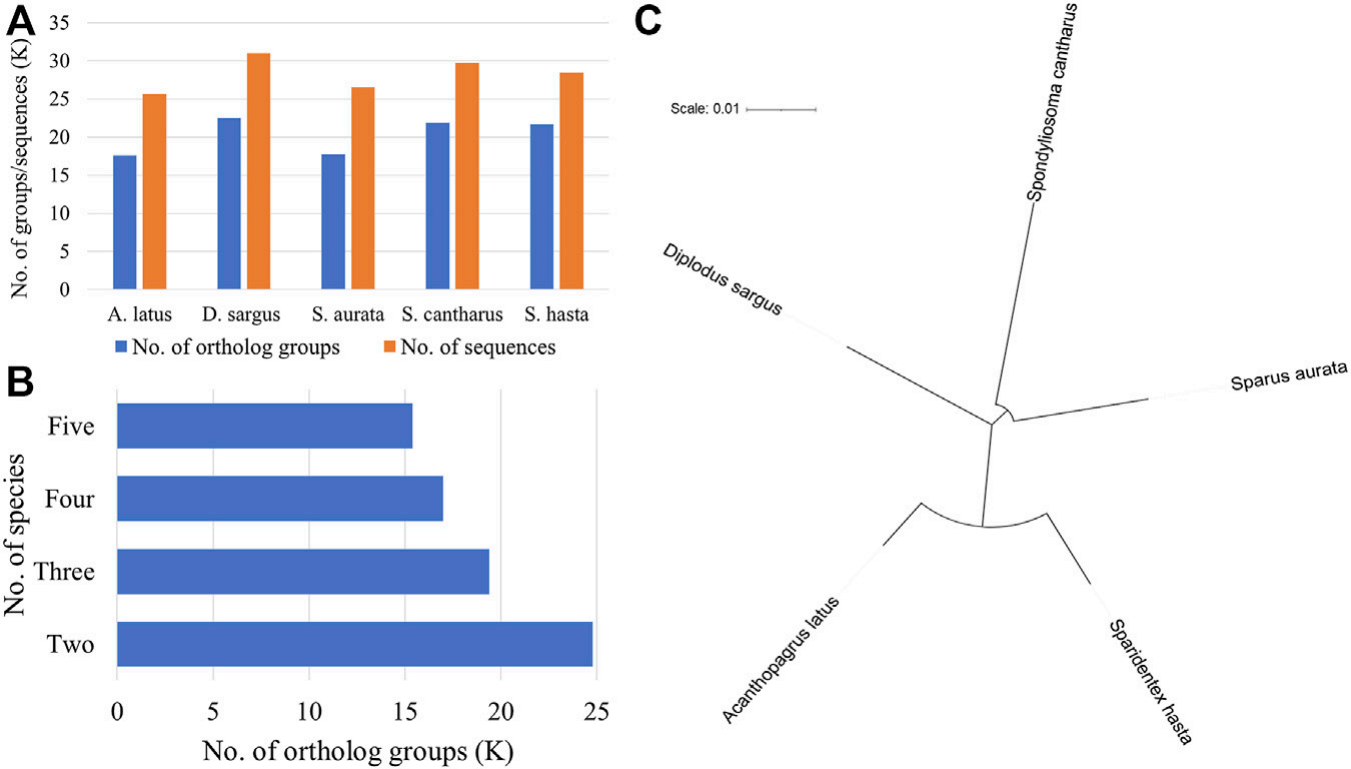
Phylogenetic analysis using protein sequences across five fish species. **(A)** No. of ortholog groups and corresponding sequences in each species. The x-axis represents the names of fish species, whereas y-axis represents the number of groups or sequences in thousand. **(B)** No. of ortholog groups shared across the five species. **(C)** Phylogenetic tree of five fish species based on single-copy orthologs.

The phylogenetic analysis was performed to understand the relationship among five fish species (*D. sargus*, *S. cantharus, S. aurata, A. latus,* and *S. hasta*) at the sequence level. The ortholog analysis revealed a total of 24,784 ortholog groups across the five species. *D. sargus* sequences were clustered into most number of ortholog groups (Figure 3A).

Further, there were 15,389 groups that were shared across all five species (Figure 3B). There were a total of 10,785 single-copy orthologs across the five species. The sequences corresponding to these ortholog groups were considered for phylogenetic tree construction. The phylogenetic tree indicated the relationship among the five seabreams and showed that *S. hasta* is closer to *A. latus*, the yellowfin seabream (Figure 3C).

## Discussion

In the current study, we assembled the draft genome sequence of Sobaity seabream, *S. hasta*, belonging to Sparidae family. The Sparidae family of fishes are economically important due to their good meat quality and good adaptability to captivity. Currently, very few species of this family have been completely sequenced at the genome level. The assembled genome size of *S. hasta* was ∼680 Mb, closely comparable to the genome of other sequenced seabreams. For instance, the genome size of *P. major* and *A. latus* was estimated to be ∼800 Mb. Our assembled genome was shown to be of high quality in terms of completeness, which was indicated based on the overall assembly statistics, such as number of Ns, read alignment and assembly completeness. The N50 statistics of our assembly was comparatively lower than that of the recently published genomes of other closely related species. For instance, the N50 of our assembly was 80 Kb, while this value for *P. major* and *A. latus* contig assembly was 2.8 and 2.6 Mb, respectively (Shin et al., 2018; Zhu et al., 2021).

The lower N50 statistics of *S. hasta* could be attributed to the unavailability of long-read/mate-pair sequences and is a limitation of the current study. Long-read sequences produced from technologies, such as PacBio and Oxford Nanopore can readily traverse the most repetitive regions and help in filling the gaps between contigs, thus increasing the length of assembled sequences, in turn improving N50 statistics (Logsdon et al., 2020). The draft genome of *S. hasta* contained a moderate number of repeats (∼22%). This was in agreement with the results from other species of the Sparidae family. The draft genome sequence of *A. latus* contained approximately 19% repeats, among which 14% were unclassified (Zhu et al., 2021). Similarly, the *S. aurata* genome contained around 20% repeats (Pauletto et al., 2018). The genome of *P. major*, however, contained a comparatively higher number of repeat sequences, which corresponded to 31% of its genome (Shin et al., 2018). Furthermore, the genome of *A. latus* contains ∼19,600 genes, whereas, the genomes of *S. aurata* and *P. major* have approximately 30,500 and 28,300 protein-coding genes, respectively. In the current study, we estimated *S. hasta* genome to contain a total of 41,201 genes (with 44,555 transcripts), slightly higher than that reported in other seabreams, and approximately, 70% of the protein sequences were significantly aligned to other protein sequences using BLAST. Further, we showed that our assembly quality was good based on the single copy orthologs and alignment of the reads to the assembled genome. The annotation results were in agreement with that of the other published seabream studies. The *S. aurata* genome contained 90% single copy genes and 91% complete BUSCO groups. The BUSCO score for *P. major* was ∼98%, whereas, in *A. latus* genome, more than 92% of BUSCO genes were identified. We detected approximately 93% of complete BUSCOs in *S. hasta* genome. The phylogenetic analysis of *S. hasta* and four other seabreams revealed a close relationship between *S. hasta* and *A. latus*.

In summary, we report the first draft assembly of *S. hasta* genome. The size of the filtered assembly was ∼686 Mb with 20,442 scaffolds. The repeat sequences were accounted for ∼22% of the genome sequence. The assembly contained a total of 44,555 transcript sequences with a mean length of 1,249 bp. Approximately 68% of the protein sequences (*n* = 30,545) had orthologs based on significant BLAST matches, and 30,473 sequences mapped to Gene Ontology annotations. Furthermore, the comparative genome analysis indicated that *S. hasta* is closer to *A. latus*, a yellowfin seabream. The current assembly provides a solid foundation for future population and conservation studies of *S. hasta* as well as for investigations of environmental adaptation in Sparidae family of fishes.

## Data Availability

The datasets presented in this study can be found in online repositories. The names of the repository/repositories and accession number(s) can be found in the article.
